# Albumin Is a Component of the Esterase Status of Human Blood Plasma

**DOI:** 10.3390/ijms241210383

**Published:** 2023-06-20

**Authors:** Daria A. Belinskaia, Polina A. Voronina, Polina I. Popova, Natalia G. Voitenko, Vladimir I. Shmurak, Mikhail A. Vovk, Tatiana I. Baranova, Anastasia A. Batalova, Ekaterina A. Korf, Pavel V. Avdonin, Richard O. Jenkins, Nikolay V. Goncharov

**Affiliations:** 1 Sechenov Institute of Evolutionary Physiology and Biochemistry, Russian Academy of Sciences, pr. Torez 44, 194223 St. Petersburg, Russia; 2 City Polyclinic No. 112, 25 Academician Baykov Str., 195427 St. Petersburg, Russia; 3 Centre for Magnetic Resonance, St. Petersburg State University, Universitetskij pr., 26, Peterhof, 198504 St. Petersburg, Russia; 4 Faculty of Biology, St. Petersburg State University, 7-9 Universitetskaya Emb., 199034 St. Petersburg, Russia; 5 Koltsov Institute of Developmental Biology, Russian Academy of Sciences, 26 Vavilova Str., 119334 Moscow, Russia; 6 Leicester School of Allied Health Sciences, De Montfort University, The Gateway, Leicester LE1 9BH, UK

**Keywords:** albumin, biomarker, cholinesterase, carboxylesterase, COVID-19, diagnostics, esterase status

## Abstract

The esterase status of blood plasma can claim to be one of the universal markers of various diseases; therefore, it deserves attention when searching for markers of the severity of COVID-19 and other infectious and non-infectious pathologies. When analyzing the esterase status of blood plasma, the esterase activity of serum albumin, which is the major protein in the blood of mammals, should not be ignored. The purpose of this study is to expand understanding of the esterase status of blood plasma and to evaluate the relationship of the esterase status, which includes information on the amount and enzymatic activity of human serum albumin (HSA), with other biochemical parameters of human blood, using the example of surviving and deceased patients with confirmed COVID-19. In experiments in vitro and in silico, the activity of human plasma and pure HSA towards various substrates was studied, and the effect of various inhibitors on this activity was tested. Then, a comparative analysis of the esterase status and a number of basic biochemical parameters of the blood plasma of healthy subjects and patients with confirmed COVID-19 was performed. Statistically significant differences have been found in esterase status and biochemical indices (including albumin levels) between healthy subjects and patients with COVID-19, as well as between surviving and deceased patients. Additional evidence has been obtained for the importance of albumin as a diagnostic marker. Of particular interest is a new index, [Urea] × [MDA] × 1000/(BChEb × [ALB]), which in the group of deceased patients was 10 times higher than in the group of survivors and 26 times higher than the value in the group of apparently healthy elderly subjects.

## 1. Introduction

A set of enzyme activities from the group of esterases constitutes the so-called “esterase status” of the organism. This term was introduced by Dr. Makhaeva in 2004 [1]. Later on, the authors suggested their perception of the esterase status as a complex biomarker of relatively short-term effects of organophosphates (OPs) on the organism [2]. However, considering that esterases play an important role in different key physiological processes, then esterase status can also reflect different functional disorders in human and animal organisms. The search for diagnostic and prognostic markers, risk assessment and probability of lethal outcome of many diseases, including COVID-19, is an urgent task of modern medicine. The esterase status of blood plasma can claim to be one of the universal markers of various diseases; therefore, it deserves attention when searching for markers of the severity of both COVID-19 and other infectious and non-infectious pathologies. Suffice it to say that the core contributor to this status is the level of acetylcholine, known not only as a mediator of neuromuscular and neuronal synapses but also as a rather powerful anti-inflammatory agent [3,4,5].

The principal esterases of human blood plasma are butyrylcholinesterase (BChE, EC 3.1.1.8) and paraoxonase 1 (PON1, EC 3.1.8.1). Activities of these enzymes in normal state and in pathology present the highest interest because they have wide substrate specificity and are involved in different vitally important processes. The concentration of other esterases—carboxylesterase (CE), neurotoxic (or neuropathic target) esterase (NTE), dimeric erythrocyte acetylcholinesterase (AChE) and monomeric unspliced AChE—is several orders of magnitude lower than that of BChE and PON1 and contributes significantly less to the esterase status [6]. Moreover, NTE and dimeric AChE are not blood plasma components but those of cell membranes of lymphocytes and erythrocytes, and therefore, these enzymes are present in blood plasma mainly as components of microvesicles [7]; their concentration and activity is unstable and generally negligible in terms of their contribution to the esterase status of blood plasma, and they precipitate during high-speed centrifugation.

It is also necessary to consider the esterase activity of serum albumin, which is not only a passive but also an active participant of pharmaco- and toxicokinetics processes. In numerous experiments, the esterase (binding of the substrate to the active site, followed by the breakdown of the complex into an enzyme and a product) or pseudoesterase (irreversible covalent binding of the substrate to a protein) activity of albumin has been shown [8,9]. There is a free thiol of cysteine Cys34 in the albumin molecule, which is able to be oxidized to sulfenic and sulfinic acid or to form disulfides with free glutathione, cysteine, homocysteine and other thiols [10]. Healthy people have about 70% reduced albumin [11], but in pathological and extreme conditions accompanied by oxidative stress, the level of oxidized albumin can increase to 70% [12]. Recent studies indicate that both a decrease in the total albumin level and an increase in the proportion of oxidized albumin are highly sensitive diagnostic markers and reliable predictors of the outcome of many common and socially significant diseases, including COVID-19 [13,14,15,16,17,18,19,20,21,22].

The albumin molecule is characterized by allosteric modulation [23], and the low catalytic efficiency of albumin is compensated by its extremely high concentration. Therefore, we believe that both the concentration of albumin in blood plasma and its (pseudo)esterase activity will depend on the state of the organism and can serve as an additional marker of the severity of pathology.

The purpose of this study is to expand the understanding of the esterase status of blood plasma and to estimate the relationship of the esterase status, which includes information on the amount and activity of serum albumin, with other biochemical parameters of human blood using the example of survivors and deceased patients with confirmed COVID-19.

## 2. Results and Discussion

### 2.1. The Effect of Sequential Addition of Inhibitors on the Esterase Status of Plasma of Healthy Humans

**Figure 1:**
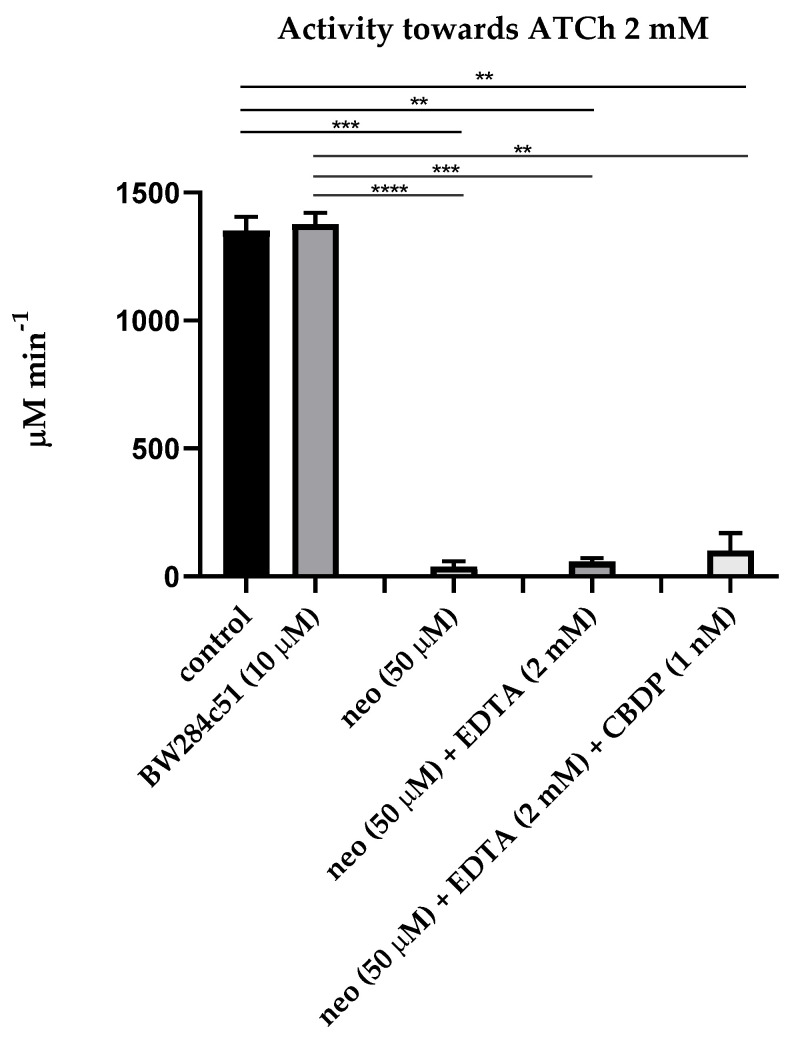
Effect of inhibitors on the pooled plasma activity towards ATCh. The plasma was used at 1:10 dilution. The control consisted of an equivalent volume of PBS 10 mM. Heparinized plasma was pooled from 10 samples of apparently healthy people. An equivalent amount of an individual sample was taken and mixed in a tube to level out individual characteristics. Abbreviations: neo, neostigmine; EDTA, ethylenediaminetetraacetic acid; CBDP, 2-(2-cresyl)-4*H*-1-3-2- benzodioxaphosphorin-2-oxide. ****, the level of significance of the change in activity *p* < 0.0001; ***, *p* < 0.001; **, *p* < 0.01 (Kruskal–Wallis test, Dunn’s post hoc test, n = 8).

**Figure 2:**
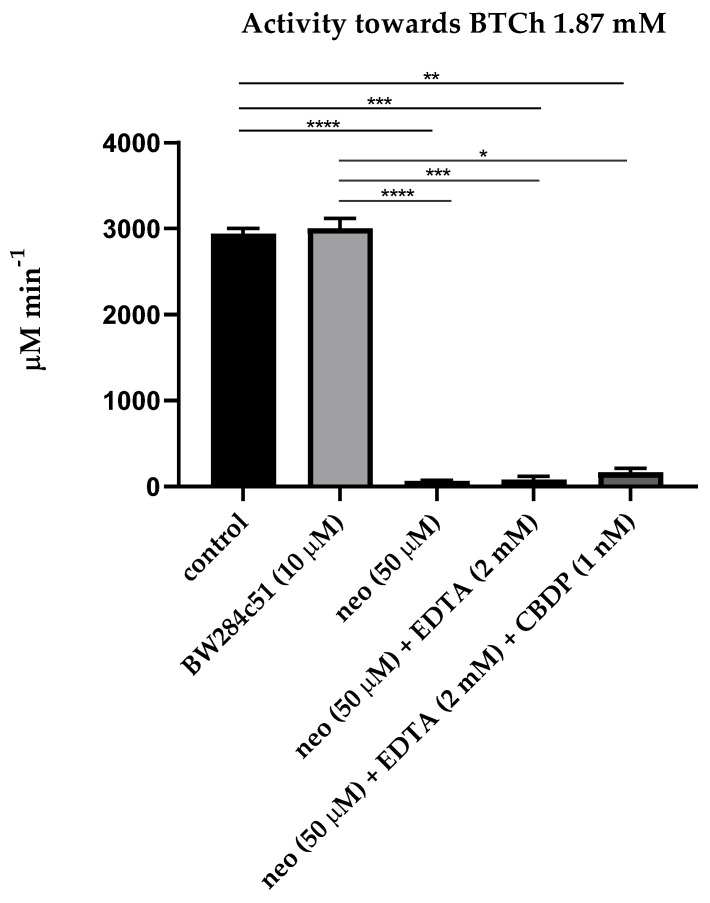
Effect of inhibitors on the pooled plasma activity towards BTCh. The plasma was used at 1:10 dilution. All legend notes are the same as for Figure 1. And *, *p* < 0.05.

The first step of our research was estimation of the influence of sequential addition of BW284c51 (specific inhibitor of AChE), neostigmine (non-specific cholinesterase inhibitor), ethylenediaminetetraacetic acid (EDTA, the PON1 inhibitor due to chelation of Ca^2+^ ions), 2-(2-cresyl)-4H-1-3-2-benzodioxaphosphorin-2-oxide (CBDP, the CE inhibitor) and sodium palmitate (a non-specific inhibitor of albumin esterase activity, along with other long-chain fatty acids [24]) on the activity of the pooled plasma of healthy subjects in relation to acetylthiocholine (ATCh, the substrate of AChE and BChE), butyrylthiocholine (BTCh, the substrate of BChE), 4-nitrophenyl acetate (NPA, the substrate of albumin and CE), phenyl acetate (PhA, the substrate of PON1) and paraoxon (POX, another substrate of PON1). Figure 1 and Figure 2 present mean values of activity of plasma with ATCh and BTCh as the substrates, with addition of different inhibitors.

As expected, the AChE inhibitor BW28c51 did not have a significant inhibitory effect on plasma activity towards ATCh and BTCh, because the activity of erythrocyte AChE in membranes or microvesicles was excluded by centrifugation of primary plasma at high rotations, and the content of monomeric (unspliced) AChE in human plasma is extremely small, approximately 8 μg/L [6]. A slight increase in the activity towards BTCh may be associated with the phenomenon of activation of BChE by cation–ammonium compounds, to which BW28c51 belongs [25,26]. Thus, we have shown that after centrifugation, there is practically no AChE in the blood plasma; therefore, the BW28c51 inhibitor of AChE is of no need for determining the activity of BChE towards ATCh and BTCh.

It was also expected that the addition of EDTA (the PON1 inhibitor) and CBDP (the CE inhibitor) after inhibition of BChE by neostigmine did not significantly reduce plasma activity towards ATCh and BTCh, since ATCh and BTCh are not specific substrates of PON1 and CE; in any case, the content of CE in human blood plasma is extremely low [6].

**Figure 3:**
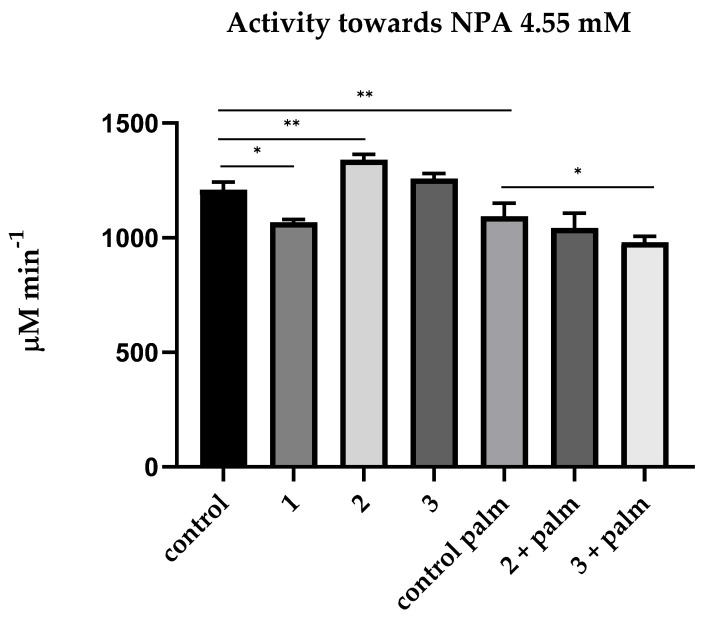
Effect of inhibitors on the pooled plasma activity towards NPA. The plasma was used at 1:10 dilution. The concentrations of the inhibitors used were calculated according to data obtained in the laboratories of O. Lockridge and E. Vilanova [6,24]. The control consisted of an equivalent volume of PBS 10 mM; 1—addition of neostigmine (50 µM); 2—addition of neostigmine (50 µM) and EDTA (2 mM); 3—addition of neostigmine (50 µM), EDTA (2 mM) and CBDP (10 nM); control palm—PBS (10 mM) with palmitate (1 mM); 2 + palm—addition of neostigmine (50 µM) and EDTA (2 mM) in buffer with palmitate (1 mM); 3 + palm—addition of neostigmine (50 µM), EDTA (2 mM) and CBDP (10 nM) in buffer with palmitate (1 mM). **, the level of significance of the change in activity *p* < 0.01; *, *p* < 0.05 (Kruskal–Wallis test, Dunn’s post hoc test, n = 4).

Then, we measured the CE activity of blood plasma using NPA as a substrate and various inhibitors. In a preliminary experiment, the effect of palmitate at concentrations of 5, 10, 20 and 30 μM on the kinetics of NPA hydrolysis by HSA and bovine serum albumin (BSA) was studied using HSA and BSA (Sigma, 10 μM) purified from fatty acids. The dynamics of hydrolysis were registered for the prestationary (1.5–4.5 min) and stationary (8.5–18.5 min) phases. No inhibition of NPA hydrolysis by palmitate was observed in the stationary phase (true esterase activity). From the averaged reaction rates, the degree of inhibition at the prestationary phase (pseudoesterase activity) was calculated relative to the hydrolysis rate of the control sample. Similar results were obtained with HSA and BSA; the data are presented in Figure S1. According to the results, palmitate should be included to buffer and separate the activity of albumin towards NPA from the activity of other esterases. Figure 3 shows the relevant mean values of the CE activity of blood plasma towards NPA.

As expected, neostigmine had a small but significant inhibitory effect on plasma activity towards NPA. In the literature, we did not find data on whether neostigmine can interact with albumin, and for this reason, an experiment was conducted in silico, in which we demonstrated that neostigmine does not affect (pseudo)esterase activity of HSA (see Section 2.4). Therefore, this effect can be explained by a non-zero activity of BChE towards NPA.

EDTA has a not very strong but significant activating effect on plasma activity towards NPA. It probably means that EDTA affects the activity of HSA towards NPA. It appears that EDTA binds in one of the albumin sites and/or chelates divalent cations bound with albumin at metal-binding sites, thus exerting an allosteric effect on the activity of another site; in addition, EDTA could affect the Kemp elimination reaction taking place in the Stern layer: the latter makes a certain contribution to the apparent kinetics of both albumin sites [27]. However, after the CBDP addition, we observed a leveling of activation caused by EDTA, which can be explained by inhibition of activity of albumin or CE, which is present in small amounts in human blood plasma. It will be demonstrated below in experiments with a spectrophotometer and NMR that CBDP in nanomolar concentrations does not affect the activity of albumin. Therefore, the inhibitory effect of CBDP is still associated with the presence of a non-zero amount of CE in human blood plasma, the source of which is obviously lysed blood cells, mainly erythrocytes [28,29]. It is necessary to use specific monoclonal antibodies to distinguish liver CES1, intestine CES2, blood plasma CES3 and erythrocyte esterase D (ESD) to definitively resolve the source. This is another point for our future research.

Incubation in a buffer containing palmitate significantly (*p* < 0.01) reduced blood plasma activity towards NPA, and the difference in the CE activity with and without palmitate can be explained by the activity of albumin. We found no effect of EDTA on hydrolysis of NPA in the presence of palmitate, which means that PON1 does not contribute to hydrolysis of this substrate in “natural” conditions, i.e., in the absence of additional calcium ions. In addition, in the presence of palmitate, CBDP has an inhibitory effect on the hydrolysis of NPA, which means the presence of a non-zero amount of CE in human blood plasma, the source of which is obviously lysed blood cells, mainly erythrocytes [28,29]. It is necessary to use specific monoclonal antibodies to distinguish liver CES1, intestine CES2, blood plasma CES3 and erythrocyte esterase D (ESD) to definitively resolve the source. This is another point for our future research.

The arylesterase activity of plasma PON1 was measured using phenyl acetate (PhA) (Table S3), while paraoxonase activity was measured using POX. Neither neostigmine nor CBDP has a significant effect on plasma activity with PhA. In additional experiments, we have shown that purified albumin does not hydrolyze PhA: the level of hydrolysis corresponds to its spontaneous hydrolysis in TRIS Ca^2+^ buffer.

While using POX (1.2 mM) as a substrate, plasma activity is much lower, corresponding with the literature data [30]. Plasma was not diluted for measurement of the activity towards POX. There were no significant differences between the activity of undiluted plasma with and without inhibitors (46.3 ± 1.3 μM min^−1^ for the control sample and 48.3 ± 1.8 μM min^−1^ for the sample with 50 μM neostigmine). HSA (600 μM) shows low activity towards POX (2.0 ± 1.4 μM min^−1^), which corresponds with the literature data [24]. Activity in a calcium-free medium (with the addition of EDTA 2 mM) is not registered for both substrates.

Thus, experiments with pooled plasma made it possible to develop an algorithm for determining the activity of BChE, PON1 and HSA in human blood plasma. It was estimated that

- The use of BW284c51, a specific inhibitor of AChE, is not required for measuring the activity of BChE towards ATCh and BTCh in plasma after centrifugation at high rotations;
- To determine the activity of albumin towards NPA, it is necessary to add neostigmine (50 μM) and EDTA, and calculate the activity as the difference between the mean values with and without palmitate;
- Measurements of PON1 activity should be carried out ideally with two substrates (POX and PhA), but to spare the time and to avoid the risk of dealing with toxic POX in clinical laboratories, applying PhA alone with the addition of neostigmine 50 μM is quite adequate.

In the following sections, to support the developed methodology of plasma esterase status measurements, firstly, we showed that esterase activity of commercial albumin preparation belongs to albumin itself but not to possible impurity esterases and, secondly, with the help of molecular modeling, explained why neostigmine does not inhibit esterase activity of HSA.

### 2.2. Effect of Ethopropazine and CBDP on the Esterase Activity of Albumin Preparation According to NMR Data

In our previous work, using NMR to detect the yield of the acetate group, we demonstrated that BSA has true esterase activity towards NPA [9]. However, the question was still open about the possible influence on the experimental result of impurities possessing esterase activity, which theoretically could be present in the commercial preparation of BSA. In the presented work, we studied the effect of the CE inhibitor CBDP and the BChE inhibitor ethopropazine on the esterase activity of commercial albumin. The purpose was to ensure that the commercial preparation of albumin does not contain contaminating proteins and that nanomolar concentrations of CBDP and ethopropazine, sufficient to inhibit the theoretically permissible amounts of CE and BChE, do not affect the enzymatic activity of BSA. Since HSA and BSA preparations used were of the same brand (Sigma, fatty-acid-free) and have similar procedure of purification, all the results obtained in BSA could be extrapolated to HSA at the current stage of research.

**Figure 4:**
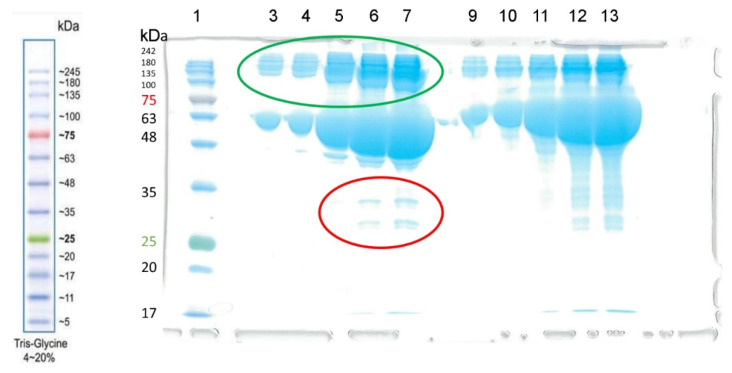
The result of electrophoresis of the BSA preparation (Sigma-Aldrich) without the addition of dithiothreitol (DTT, columns 3–7) and with the addition of DTT (columns 9–13). The concentration of the BSA solution was 4 mg/mL. Electrophoretic line 1 was loaded with 3 µL of BLUelf Prestained Protein Ladder (GeneDirex). Lines 3–7 and 9–13 were loaded with 1, 2, 5, 10 and 15 µL of BSA solution, respectively. Albumin monomers and their aggregates (dimers, tetramers) are marked in green. Contaminant proteins are marked in red.

At the first stage, we performed electrophoresis of the BSA preparation used in this work. The result is shown in Figure 4. As can be seen, contaminant proteins were found (marked in red) in the last two lines (6 and 7) in the experiment without the addition of DTT with a maximum load (10 and 15 µL of a solution with a concentration of 4 mg/mL BSA, respectively, corresponding to 40 and 60 µg of protein). The detection limit for the dye used (bromophenol blue) is approximately 8 ng of protein. Assuming that line 6 contains two impurities of approximately 10 ng each, the relative content of each impurity is approximately 0.025%.

Judging by the mass, it is most likely that these proteins are globulins. However, it must be ensured that the BSA preparation does not contain other esterase impurities hidden by dimers or oligomers. In addition to albumin, BChE (tetramer with m.w. 440 kDa) and CE (monomers of about 60 kDa tending to form trimers with m.w. 182 kDa) can hydrolyze NPA in blood plasma. We excluded the activity of paraoxonase PON1, AChE and neuropathy target esterase (NTE), since PON1 is not active in a calcium-free environment, erythrocyte AChE is associated with erythrocyte membranes, and NTE is associated with lymphocyte and erythrocyte membranes [2]. Monomeric (non-spliced) AChE is also present in the blood plasma, but in bovines and humans, its concentration is very low, approximately 8 µg L^−1^, which is approximately seven orders of magnitude less than the concentration of albumin. To ensure that contaminant proteins do not contribute to the esterase activity of BSA, we used NMR to analyze the hydrolysis of NPA by BSA in the presence of ethopropazine and CBDP, which are BChE and CE inhibitors, respectively.

The concentration of BSA in the NMR experiment was 360 µM. It is known that in human and bovine blood plasma the concentration of BChE is 10,000 times (four orders of magnitude) lower than the concentration of albumin, and the concentration of CE is at least an order of magnitude lower than the concentration of BChE [6]. Thus, even in a poorly purified BSA preparation with a concentration of 360 μM, the concentration of BChE is a maximum of 36 nM, and the concentration of CE is a maximum of 3.6 nM. In the presented experiment, the concentrations of inhibitors added to the BSA samples were 240 nM and 24 nM for ethopropazine and CBDP, respectively. That is, the ratio with the concentrations of potential esterases was at least 6:1.

**Figure 5:**
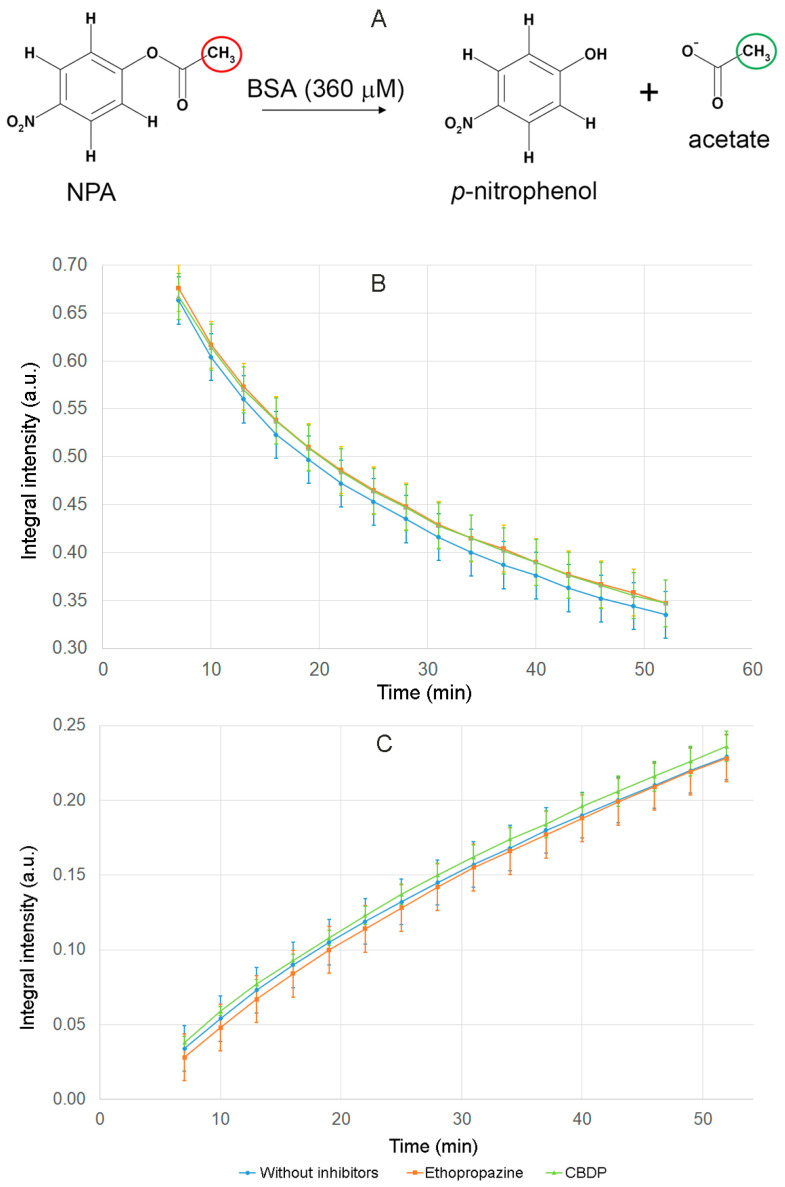
Hydrolysis of NPA in the presence of BSA and BChE and CE inhibitors (ethopropazine and CBDP) according to NMR data. (**A**) schematic representation of the hydrolysis of NPA by BSA; (**B**) time dependence of the relative integral intensity (I_rel_) of the signal of the hydrogens of NPA methyl group in the reaction mixture in the presence of BSA, ethopropazine and CBDP; (**C**) time dependence of I_rel_ of the signal of the hydrogens of the free-acetate group in the reaction mixture in the presence of BSA, ethopropazine and CBDP. I_rel_ was calculated as the ratio of the integrated signal intensity of the substrate or product to the integrated signal intensity of the internal standard DMSO d6.

The experiment was repeated three times. The result is shown in Figure 5. The schematic reaction of NPA hydrolysis by BSA is shown in Figure 5A. The methyl group of NPA is indicated by red color in Figure 5A; we used the relative integral intensity of NMR signal of the hydrogens of this group to estimate the loss of the substrate during the reaction. The methyl group of acetate is indicated by green color in Figure 5A; we used the relative integral intensity of NMR signal of the hydrogens of this group to estimate the yield of the product of the true esterase activity of BSA.

The dependence of the relative integral intensity of the signals of the methyl group of NPA (Figure 5B) and the free-acetate group (Figure 5C) on time was plotted. The signal intensity of the substrate and reaction product was calculated relative to the integral signal intensity of the internal standard DMSO d6. The operation of the spectrometer is such that 7–10 min pass between the addition of the substrate to the enzyme and the detection of the first spectrum. By this time, sites Sudlow II of BSA molecules are already completely acetylated [31,32]; therefore, the spectra obtained reflect the kinetics of an exclusively true esterase reaction of albumin, which is, as we believe, is determined by the work of Sudlow site I.

According to the data obtained, CE and BChE inhibitors do not affect the rate of substrate loss and the rate of increase in the esterase reaction product. On the basis of the results obtained, we concluded that, firstly, the hydrolysis of NPA in the presence of BSA is performed by albumin but not by possible impurity proteins, and secondly, nanomolar concentrations of CBDP and ethopropazine do not affect the esterase activity of physiological concentrations of albumin.

### 2.3. Effect of Ethopropazine and CBDP on the (Pseudo)esterase Activity of Albumin Preparation According to Spectrophotometric Data

**Figure 6:**
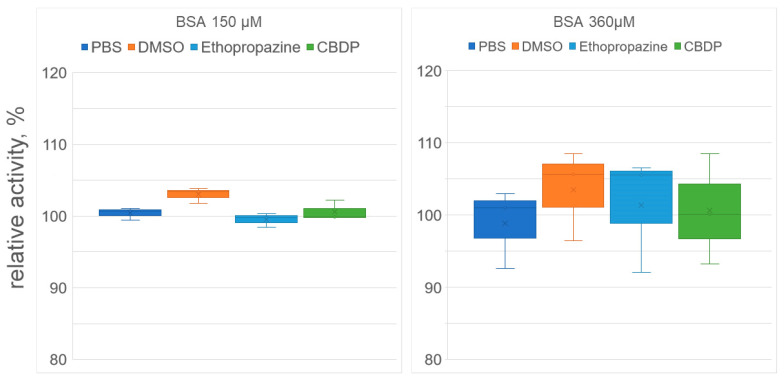
Effect of inhibitors on the (pseudo)esterase activity of BSA. The concentrations of BSA, ethopropazine, CBDP and NPA in the reaction mixture were 150, 2.5, 0.025 and 3000 µM in experiment #1 and 360, 6, 0.06 and 7200 µM in experiment #2, respectively. The reaction mixtures of BSA and NPA in phosphate buffer (PBS) and in PBS with the addition of DMSO (volume equivalent to the volume of the inhibitor solutions) were used as the controls. No significant differences were found.

At the next stage, we used spectrophotometry to study how ethopropazine and CBDP affect the yield of *p*-nitrophenol when NPA interacts with BSA. The advantage of this approach to studying the enzymatic activity of albumin is that the yield of the product can be observed in the first seconds of the reaction (in contrast to NMR, when the first spectrum can be obtained several minutes after start of the reaction). The disadvantage of the spectrophotometric approach is that it is impossible to separate the pseudoesterase activity of albumin from its true esterase activity, since nitrophenol is the product of both esterase and pseudoesterase activities. The experiment was performed with two concentrations of BSA, 150 µM and 360 µM. The results of the experiment are shown in Figure 6.

According to the data obtained, CBDP and ethopropazine had no significant effect on the rate of nitrophenol release, both for 150 μM and 360 μM BSA concentrations. As noted above (see Section 2.2), using the NMR experiment, we showed that CBDP and ethopropazine do not affect the kinetics of the true esterase activity of albumin (which we believe takes place at Sudlow site I). Using spectrophotometry, we observed the yield of nitrophenol in the first minutes of the reaction, which is due to the work of the pseudoesterase activity site Sudlow II. Thus, the combination of NMR and spectrophotometric data allows us to state that CBDP and ethopropazine at the concentrations in which they inhibit CE and BChE do not affect the activity of either Sudlow site I or Sudlow site II. The question of the possibility of the participation of impurity proteins in the BSA preparation in the hydrolysis of NPA was also closed.

It is interesting to note that DMSO had some activating effect, which, however, also turned out to be statistically insignificant. It is known that a certain contribution to the apparent kinetics of both sites of albumin is made by the so-called Kemp elimination, which is a prototypical reaction of proton decoupling from carbon, which probably occurs in the Stern layer, at the interface between the micelle head or protein surface and water, so that a significant acceleration of the reaction can be achieved regardless of the spatial arrangement of the substrate [33,34,35]. We believe that DMSO can affect the distribution of charges on the surface of albumin and, thus, the nonspecific hydrolysis of NPA occurring in the Stern layer.

### 2.4. Interaction of Neostigmine with Human Serum Albumin According to Molecular Modeling Data

It was previously shown using MALDI-MS that when interacting with NPA, HSA can be acetylated at 82 amino acid residues (59 lysines, 10 serines, 8 threonines, 4 tyrosines and 1 aspartate) [31]. Among sites of acetylation of HSA, NPA has the highest affinity for Sudlow site II with the catalytically active Tyr411, which is the first amino acid to be acetylated. According to Lockridge et al. [31], when albumin interacts with NPA, Tyr411 is acetylated within 5 min, while the formation of adducts with other amino acids requires at least 30 min.

Previously we applied proton NMR technology to detect the yield of the acetate group and showed that albumin had true esterase activity towards NPA at a site different from Sudlow site II [9]. Using molecular modeling methods, we substantiated that Sudlow site I with catalytic Tyr150 is highly likely responsible for the true esterase activity of albumin [9,32]. Thus, Sudlow site I with catalytic Tyr150 and Sudlow site II with catalytically active Tyr411 are the main sites of albumin hydrolytic activity towards NPA.

To assess whether the cholinesterase inhibitor neostigmine can have an inhibitory effect on the hydrolytic activity of albumin, we have studied the interaction of neostigmine with Sudlow sites I and II of HSA using molecular modeling methods. Molecular docking of neostigmine to Sudlow sites was performed, and then the conformational changes in the obtained complexes were studied by molecular dynamics (MD).

#### 2.4.1. Interaction of Neostigmine with Sudlow Sites I and II of HSA

Detailed description of neostigmine interaction with HSA is presented in the Supplementary Materials. Shortly, according to MD simulation, neostigmine binds in that part of Sudlow site I that corresponds to fatty-acid binding site FA7 (Figure S2). Thus, the central zone of Sudlow site I with catalytic Tyr150 is partially available for NPA binding. Whether NPA can bind to Sudlow site I in the presence of neostigmine is discussed below. According to MD simulation, neostigmine is not a ligand of Sudlow site II and cannot inhibit pseudoesterase hydrolysis of NPA at this site (Figure S3).

In the next step, we examined whether the binding of neostigmine to Sudlow site I could affect the interaction of NPA with Sudlow sites. We performed molecular docking of NPA into Sudlow sites I and II of free HSA and HSA with neostigmine bound in Sudlow site I. As a model for free albumin, we used the crystal structure of HSA picked from the Protein Data Bank (PDB), which then was optimized by the energy minimization method. As a model for the complex HSA-neostigmine, we used the structure obtained by a 100 ns MD simulation with neostigmine at Sudlow site I. The details of the model preparation and molecular docking procedure are described in Section 3.7.1.

#### 2.4.2. Effect of Neostigmine on the Interaction of NPA with Sudlow Site I of HSA

**Figure 7:**
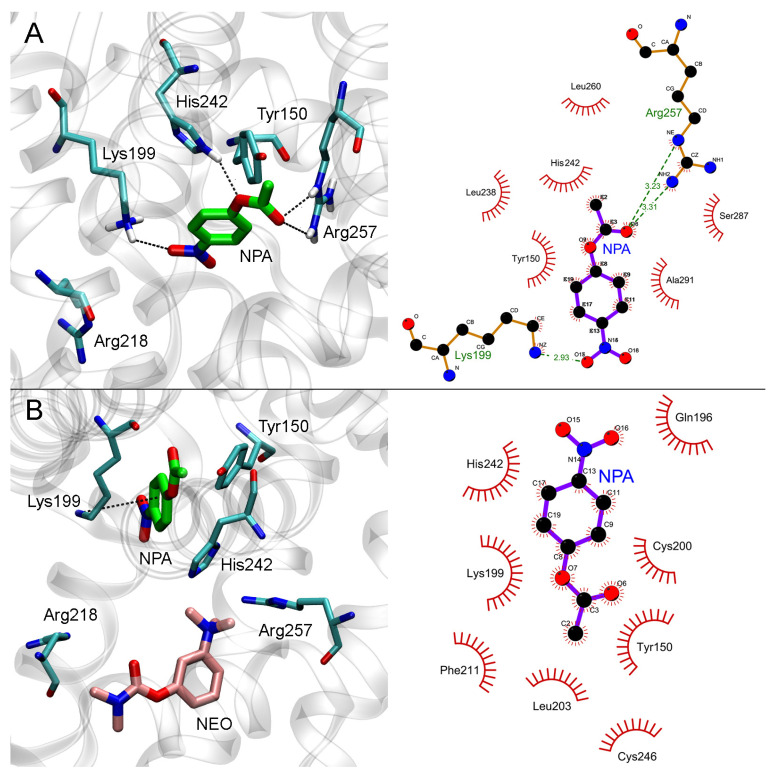
Interaction of NPA with Sudlow site I of HSA in the absence (**A**) and in the presence (**B**) of neostigmine (NEO) in Sudlow site I. Three- and two-dimensional views of NPA binding modes inside Sudlow site I are shown in the left and right panels, respectively. Non-essential hydrogens are omitted for clarity.

The results of molecular docking of NPA into Sudlow site I of HSA with or without neostigmine are shown in Figure 7.

In the case of free albumin, the carboxyl oxygen atom of NPA forms a hydrogen bond with the guanidine group of Arg257, and the nitro group of NPA forms a hydrogen bond with the NH_3_-group of Lys199 (Figure 7A). The hydrogen atom of the NH-group of His242 imidazole ring forms a hydrogen bond with the etheric oxygen atom of NPA. The distance between the oxygen atom of the hydroxyl group of Tyr150 and the carboxyl carbon atom of NPA is 3.9 Å, which is sufficient for the nucleophilic attack of the tyrosine hydroxyl on the carbon atom of NPA and the disruption of the ester bond.

When neostigmine interacts with Sudlow site I of HSA, the trimethylammonium group of the inhibitor partially occupies the space between Arg257 and His242 involved in NPA binding (Figure 7B). Nevertheless, according to molecular docking data, an NPA molecule in Sudlow site I can bind in such a position that the nucleophilic attack of a Tyr150 hydroxyl oxygen atom on an NPA carboxyl carbon atom is possible. In the productive conformation (Figure 7B), the distance between the oxygen atom of the hydroxyl group of Tyr150 and the carboxyl carbon atom of NPA is 3.1 Å. Thus, the binding of neostigmine in Sudlow site I does not affect catalytic Tyr150, and we believe that NPA hydrolysis at this site is still possible.

#### 2.4.3. Effect of Neostigmine on the Interaction of NPA with Sudlow Site II of HSA

**Figure 8:**
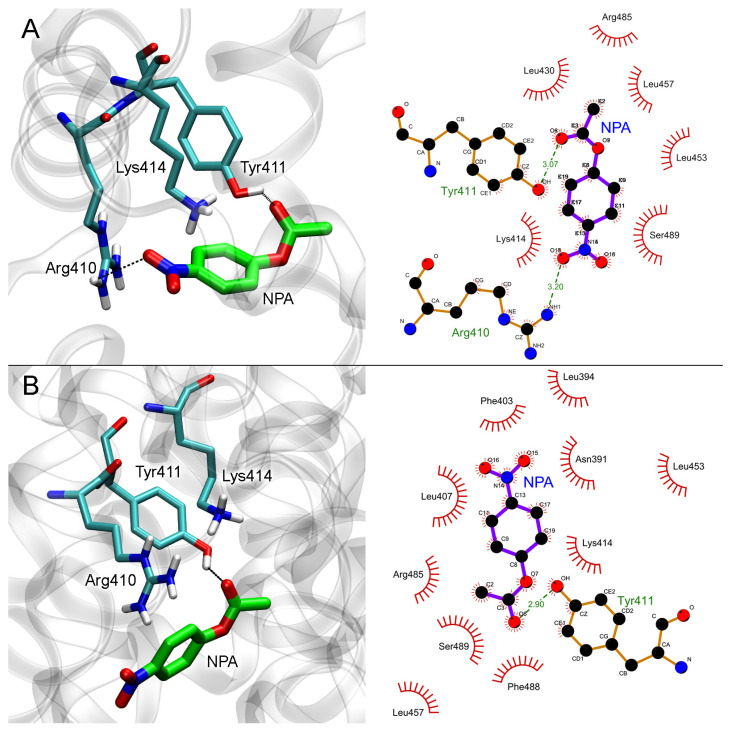
Interaction of NPA with Sudlow site II of HSA in the absence (**A**) and in the presence (**B**) of neostigmine (NEO) in Sudlow site I. Three- and two-dimensional views of NPA binding modes inside Sudlow site II are shown in the left and right panels, respectively. Non-essential hydrogens are omitted for clarity.

The results of molecular docking of NPA into Sudlow site II of free HSA and HSA with neostigmine bound in Sudlow site I are shown in Figure 8.

In the case of free albumin (Figure 8A), the distance between the oxygen atom of the hydroxyl group of Tyr411 and the carboxyl carbon atom of NPA is 3.5 Å. The binding of neostigmine in Sudlow site I does not allosterically affect Sudlow site II, while NPA binds in the same configuration in both free HSA and neostigmine–HSA complex (Figure 8B). The distance between the oxygen atom of the hydroxyl group of Tyr411 and the carboxyl carbon atom of NPA in NPA–neostigmine–HSA complex is 3.3 Å.

Therefore, according to the data of computational experiments, neostigmine does not inhibit the hydrolytic activity of albumin towards NPA. Molecular modeling data explain the result of the in vitro experiments (according to which, neostigmine does not affect the hydrolysis of NPA by albumin) and reveal the details of the mechanism of interaction of neostigmine with the ligand-binding sites of albumin.

Thus, molecular modeling is a convenient tool to support and explain the results of in vitro inhibitor analysis of blood plasma. Of course, in silico inhibitory analysis makes sense only for those enzyme–inhibitor pairs whose concentrations are comparable in in vitro experiment.

### 2.5. Esterase Status of Human Plasma in Normal and Pathological Conditions

Having tested the methodology for determining the activity of various esterases in blood plasma, we performed a comparative analysis of the esterase status and a number of basic biochemical parameters of the blood plasma of healthy subjects and patients with confirmed COVID-19. For the study, the blood of the following groups of patients was used: elderly conditionally healthy people aged 60–70 (elderly people, or elderlies, n = 9), healthy middle-aged people (30–50 years old, n = 12) practicing open-water swimming (swimmers), surviving patients with COVID-19 (survivors, n = 19) and deceased patients with COVID-19 (non-survivors, n = 9). The age of the last two groups was 60–70 years, i.e., they were elderly patients.

#### 2.5.1. Esterase Status of Healthy Subjects: Selection of the Control Group

First, we determined whether it was correct to combine the groups “elderlies” and “swimmers” into one control group of healthy subjects. We compared the esterase status and other biochemical parameters in these groups. The results are presented in Table 1.

From the entire spectrum of clinical biochemistry, Table 1 includes the following parameters: the concentrations of urea, creatinine, albumin and total protein. Significant differences between the groups were estimated in creatinine concentration (1.5 times higher in swimmers, *p* < 0.0001) and in total protein (8% higher in swimmers, *p* < 0.01). There were no significant differences between the groups in the content of albumin and urea. When evaluating the albumin-to-total-protein ratio, we revealed expected differences between the groups. The concentration of MDA in the blood plasma of swimmers is significantly (*p* < 0.05) lower by 27%.

BChE activity towards ATCh and BTCh also differs significantly between groups. BChE activity towards ATCh is higher by 24% (*p* < 0.05) in the group of swimmers. BChE activity towards BTCh, measured without the use of inhibitors, is also higher in the group of swimmers by 40% (*p* < 0.01). There was also a trend towards an increase in the esterase activity of albumin and PON1 in swimmers, but without statistical significance.

At the next stage, we calculated the combined indices of blood plasma characteristics of apparently healthy elderly people and middle-aged swimmers. The [urea]/[ALB] ratio does not differ in these groups of healthy people with different levels of physical fitness, while pronounced differences are observed in the ratio of BChE activities to [ALB] and [MDA], as well as in the ratio [Urine] × [MDA] × 1000/(BChEb × [ALB]). Thus, the index BChEa/[ALB] (BChE-activity-towards-ATCh-to-concentration-of-albumin ratio) in the group of swimmers is higher by 22% (*p* < 0.01), and the index BChEb/[ALB] (BChE-activity-towards-BTCh-to-concentration-of-albumin ratio) differs by 36% (*p* < 0.01). When using the concentration of MDA instead of [ALB] in the index, we see even more pronounced differences: an increase by 74% and 98% (for BChEa (*p* < 0.01) and BChEb activity (*p* < 0.05), respectively). The index [Urea] × [MDA]/[ALB] is significantly decreased in the group of swimmers by almost two times, and finally, the index [Urea] × [MDA] × 1000/(BChEb × [ALB]) is decreased by as much as three times.

As it was expected and can be seen from the presented data, there are statistically significant differences between “elderlies” and “swimmers” for a number of indices. This does not allow these groups to be combined into a common control group but allows the formation of two independent control groups: “elderlies” and “swimmers”.

**Figure 9:**
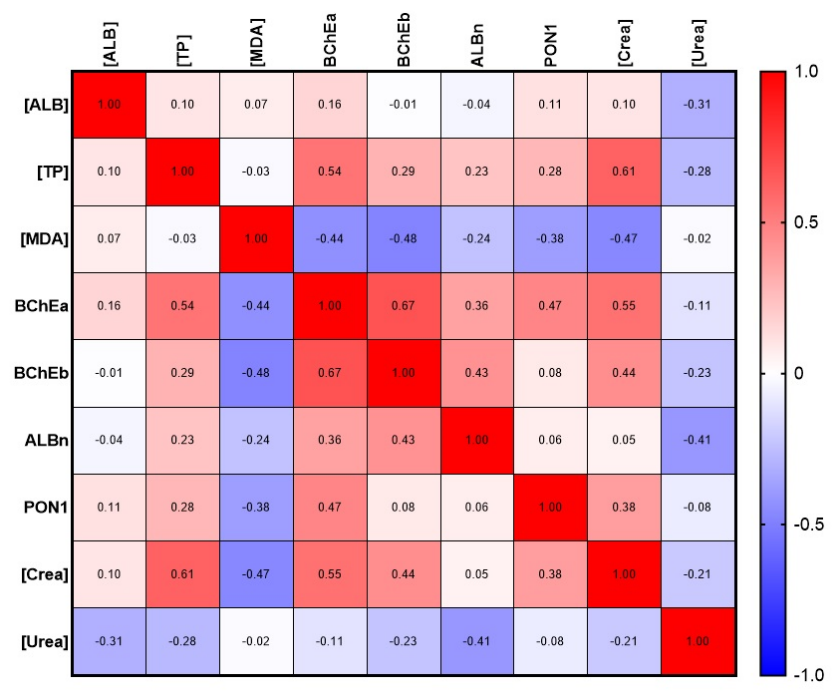
Albumin and esterase status correlation matrix with selected biochemical parameters. [ALB], concentration of albumin; ALBn, activity of albumin towards NPA; BChEa, activity of BChE towards ATCh; BChEb, activity of BChE towards BTCh; [Crea], concentration of creatinine; [MDA], concentration of malondialdehyde; PON1, activity of PON1; [TP], total protein; [Urea], concentration of urea.

Next, we calculated the correlation dependence of albumin and different esterase status parameters using Spearman’s rank correlation (Figure 9). Correlation analysis of data from the control groups (elderlies and swimmers) did not reveal any statistically significant relationship between albumin concentration and esterase status parameters. A weak correlation without statistical significance was found between the esterase activity of albumin towards NPA (ALBn) and BChEb (r = 0.43), as well as a negative relationship with urea concentration (r = −0.41). At the same time, BChEa statistically significantly (*p* < 0.05) correlates with [TP] (r = 0.54) and creatinine concentration (*p* < 0.01, r = 0.55); the correlation with PON1 activity is less pronounced (*p* < 0.05, r = 0.47). The most stable correlation was noted between BChEa and BChEb (*p* < 0.001, r = 0.67). A weak negative correlation was found between MDA concentration and BChEb (*p* < 0.05, r = −0.48). A stable correlation was found between the concentration of TP and creatinine (*p* < 0.01, r = 0.61).

Noteworthy is the lack of correlation between plasma albumin concentration and albumin activity towards NPA, as well as between albumin activity or concentration, on one hand, and PON1 activity towards PhAc, on the other hand. The latter means, in particular, that functionally these two proteins do not compensate and do not replace each other, despite the common carboxylesterase activity. In addition, the lack of correlation between the concentration and activity of albumin indicates its possible modifications (oxidation, glycation, crowding effects, interaction with fatty acids, bilirubin, polyphenols, etc.) that change its activity at the same concentration.

#### 2.5.2. Comparative Analysis of Esterase Status of Survivors and Deceased Patients with COVID-19

Patients with available data on the concentration of albumin in the blood were chosen for estimation of esterase status. The samples were divided into two groups, “survivors” and “non-survivors”, depending on outcome (Table 2). The selected set for estimation of the esterase status was 19 patients from the “survivors” and 10 from the “non-survivors”, both subsets aged 60–70 years old. The formed groups of patients do not statistically differ in age. According to the gender composition in the group of survivors, men made up 58% of the group (11 out of 19), while men made up 70% (7 out of 10) in the group of the deceased; however, there is no statistical significance by Chi-square.

The concentration of albumin is statistically significantly decreased by 11% in the “non-survivors” group compared with the survivors. The concentration of total protein tends to decrease, but these differences do not reach statistical significance. While calculating the albumin/total protein index, there is also a downward trend (0.05 < *p* < 0.1) in the deceased group. The concentration of MDA in the blood plasma of deceased patients is 1.5 times higher than in the plasma of survivors. BChEa was statistically significantly lower by 29% compared to survivors. The activity of BChEb is also significantly reduced in the blood plasma of the deceased, by 40% (*p* < 0.0001).

BChE presents in organisms in common concentrations of about 680 nM and can be found out in blood plasma, liver, brain, muscles, saliva, kidneys, heart, mucous membrane of blood vessels, skin, colon, small intestine, spleen and lungs [36]. One of the most important abilities of BChE is the detoxification of organophosphates by forming a covalent bond with them. Due to that fact that esters containing a positively charged fragment are the best substrates for BChE, this enzyme determines the period of action of pharmaceuticals with anesthetic and muscle relaxant effects, such as novocaine, mivacurium chloride and succinylcholine [36]. Furthermore, this enzyme directly involves (or is involved) in the processes of digestion and obesity, because one of the endogenous substrates of BChE is ghrelin, a hormone that causes hunger [37]. A decrease in the level of ghrelin in plasma was noted in animals with high levels of BChE in the blood. BChE converts ghrelin into deacyl ghrelin, which has reduced affinity for the growth hormone secretagogue receptor 1 (GHSR1), also known as ghrelin receptor. The injection of animals with iso-OMPA, a specific BChE inhibitor, returned ghrelin levels to the previous level. A decrease in the activity of this enzyme leads to excessive weight gain. The same was found in animals receiving low doses of chlorpyrifos, a BChE inhibitor [37]. BChE is included in the processes of regulating fat metabolism, and its activity correlates significantly with various indicators of obesity, such as waist circumference, liver fat content, visceral fat area (VFA), subcutaneous fat area (SFA), body mass index (BMI), serum lipid status and the degree of insulin resistance [38]. Liver diseases (cirrhosis and chronic hepatitis) are also associated with increased BChE activity [39]. As for the healthy elderly population, no correlation was found between BChE levels and old age, which suggests that age itself is not associated with a decrease in BChE activity. On the other hand, elderly people are often subjects for different drug treatment, and esterases are usually among the first enzymes to participate in drug metabolism [40]. It is known that BChE deficiency is mainly caused by mutations in the BChE gene (OMIM 177400). Currently, more than 70 natural mutations in human BChE have been registered. They affect the catalytic activity and/or expression of the protein. However, the atypical variant (rs1799807) is most often associated with prolonged apnea [41]. In addition to the genetic factor, the reasons for the decrease in BChE activity are old age, pregnancy, severe liver diseases, burn injuries and drug interactions [36]. However, to the best of our knowledge, a radical decrease in the activity of BChE in COVID-19 disease has not been previously described in the scientific literature.

The esterase activity of albumin and the activity of PON1 in blood plasma does not undergo significant changes in the groups under consideration. With a decrease in the concentration of albumin, the preservation of its activity means that its conformation changes under the influence of certain factors, and the specific activity increases. Further, in order to increase the diagnostic significance, presented ratios were calculated: tenfold urea concentration to albumin concentration, [Urea] × 10/[ALB]; albumin concentration to MDA concentration, [ALB]/[MDA]; BChE activity towards ATCh to albumin concentration, BChEa/[ALB]; BChE activity towards BTCh to albumin concentration, BChEb/[ALB]; the product of MDA and creatinine concentrations to albumin concentration, [Creatinine] × [MDA]/[ALB]; the product of MDA and urea concentrations to albumin concentration, [Urea] × [MDA]/[ALB]; BChE activity towards ATCh to MDA concentration, BChEa/[MDA]; BChE activity towards BTCh to MDA concentration, BChEb/[MDA]; and finally the product of MDA and urea concentrations to the product of BChE activity towards BTCh and albumin concentration, [Urea] × [MDA]/(BChEb × [ALB]).

In the group of non-survivors, the index [Urea] × 10/[ALB] is more than four times higher than in the group of survivors (*p* < 0.0001). When the MDA concentration index is introduced into the numerator, the values in the groups differ by almost six times with high statistical significance (*p* < 0.001). When this index is supplemented with another indicator—BChEb activity in the denominator—the differences between the groups reach a maximum value of 8.9 times (*p* < 0.0001). A number of other indices calculated by us are of intermediate importance due to insufficiently high significance and/or magnitude of differences. Thus, the index [Urea] × [MDA]/(BChEb × [ALB]) can claim to take a place among the most promising indices proposed to date, among which there is none that includes the activity of BChE and at the same time demonstrates such striking differences at the stage of admission to the clinic between survivors and those who died during treatment from COVID-19. At the same time, it should be mentioned that most of the proposed indices include albumin in the denominator: LDH/[ALB], [Lact]/[ALB], [Fibr]/[ALB], [Urea]/[ALB], and [CRP]/[ALB] [42,43,44,45,46,47,48,49,50,51]. Importantly, correlation analysis independent of age and concomitant pathology showed that the ratio of [CRP]/[ALB] negatively correlates with oxygen saturation (SO_2_), spirometric indices (FEV1, forced expiratory volume in the first second, and FVC, forced volume capacity) and lung diffusion values (DLCO, carbon monoxide diffusion capacity) [52].

It is necessary to underline the availability and relative low cost of the components of the index proposed by us for numerous laboratories that do not have the ability to conduct expensive analyses in order to determine indicators that are certainly elevated during a cytokine storm (ferritin, procalcitonin, troponin, calprotectin, IL-6, etc. [53,54]).

Interestingly, it was not possible to detect statistically significant differences between the groups when using the [Creatine] × [MDA]/[ALB] index. This formally “negative” result seems to be no less important than many “positive” results, taking into account the high statistical significance of the differences between the groups of those indices in which the “renal” marker is urea. This means that the critical pathogenetic factor is not so much kidney damage as enhanced urea synthesis, and not only in the classical way in the urea cycle in hepatocytes but also in endothelial cells from L-arginine due to the activity of arginases I and II, depriving eNOS of the substrate for the synthesis of NO [55,56,57,58]. An increase in the activity of endothelial arginases was actually noted in COVID-19 [59].

**Figure 10:**
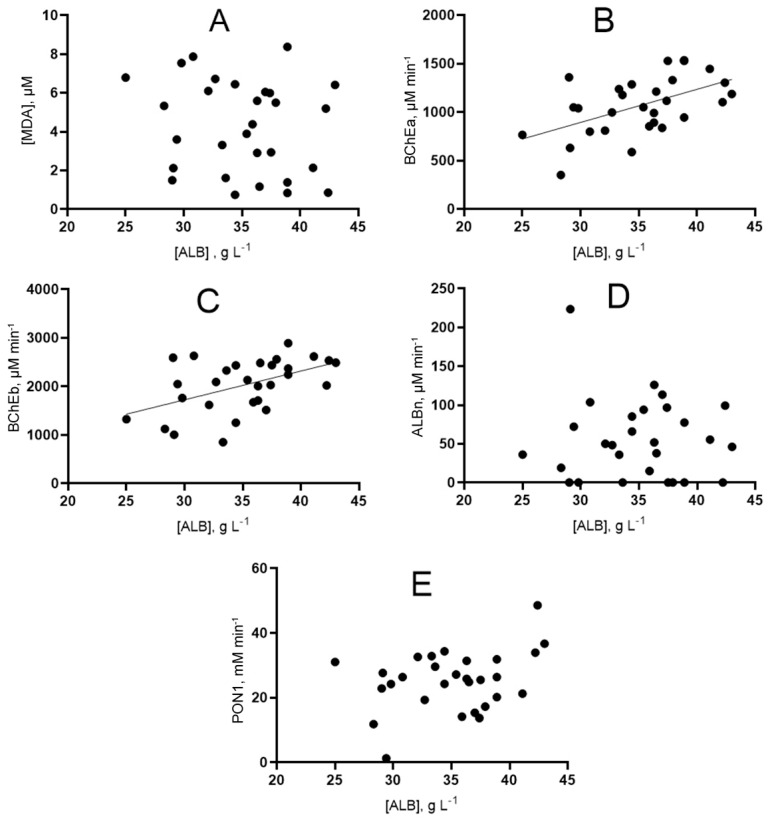
Graphical representation of the correlation relationship between the concentration of albumin and indicators of the esterase status of patients. (**A**) dependence of albumin concentration on MDA concentration; (**B**) dependence of albumin concentration on BChE activity towards ATCh; (**C**) dependence of albumin concentration on BChE activity towards BTCh; (**D**) dependence of albumin concentration on albumin esterase activity; (**E**) dependence of albumin concentration on PON1 activity.

The correlation dependence of albumin and esterase status indicators was analyzed regardless of the outcome (the entire array was taken) using Spearman’s rank correlation (Figure 10). The values of Spearman’s rank correlation coefficients and the statistical significance of the identified relationships are presented in Table S4.

The study of correlation dependencies separately among survivors and non-survivors did not reveal new correlational relationships; moreover, the correlations found in individual samples are not preserved.

**Figure 11:**
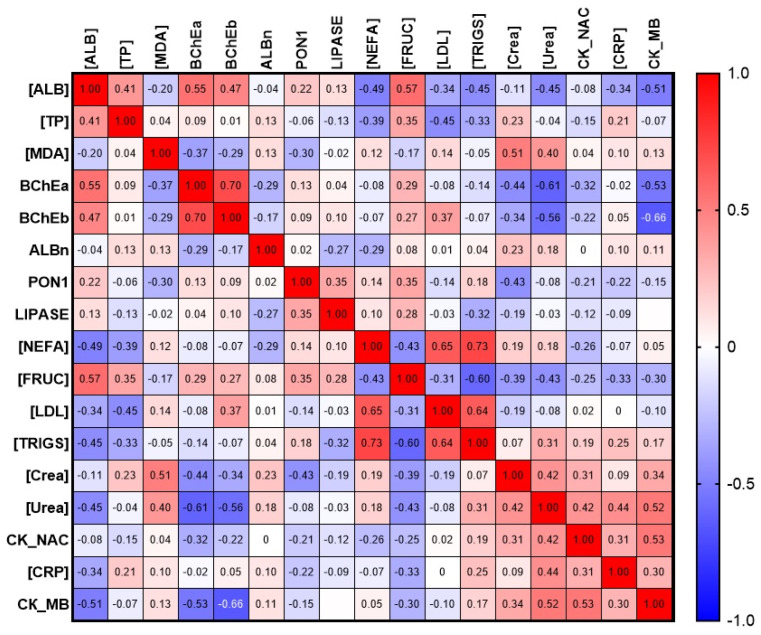
Matrix of correlation of albumin and esterase profile parameters with selected biochemical parameters. [ALB], concentration of albumin; ALBn, activity of albumin towards NPA; BChEa, activity of BChE towards ATCh; BChEb, activity of BChE towards BTCh; [CRP], concentration of C-reactive protein; CK_MB, activity of creatine kinase of myocardial band; CK_NAC, activity of NAC-activated creatine kinase; [Crea], concentration of creatinine; [FRUC], concentration of fructosamine; [LDL], concentration of low-density lipoprotein; LIPASE, activity of lipase; [MDA], concentration of malondialdehyde; [NEFA], concentration of non-esterified fatty acids; PON1, activity of PON1; [TP], concentration of total protein; [TRIGS], concentration of triglycerides; [Urea], concentration of urea.

Additionally, the correlation of esterase status and albumin with other biochemical parameters was evaluated. Preliminary results are presented in the form of a matrix in Figure 11.

#### 2.5.3. Comparative Analysis of Esterase Status of Healthy Subjects and Patients with COVID-19

We carried out comparative analysis of esterase status of healthy subjects, described in Section 2.5.1, and patients with COVID-19. The results of the comparison of the control with survivors and those who died from coronavirus infection are presented in Table 3.

The level of albumin in the survivors and non-survivors was statistically significantly lower by 18 and 27%, respectively, relative to the control, whereas for the total protein, a statistically significant decrease of 11% was noted only for the group of deceased patients. This indicates, firstly, the critical role of albumin in the outcome of the disease and the high significance of this indicator for future risk stratification, which is confirmed by numerous data from other researchers [18,22,46,53], and secondly, the preservation of the protein-synthesizing function of the liver in patients with a negative prognosis, and even some increase in it, mainly due to the synthesis of CRP when stimulated by pro-inflammatory cytokines [60]. The esterase activity of albumin does not undergo significant changes, while the activity of PON1 in both groups of COVID-19 patients, regardless of the outcome, significantly decreases by 35%.

The concentration of MDA in the blood plasma of the patients is significantly higher than in the control group. In the group of survivors, the median MDA value is three times higher, and in the group of non-survivors, it is five times higher (*p* < 0.05 and *p* < 0.001, respectively). The activity of BChE towards ATCh and BTCh is significantly decreased in the deceased (non-survivors) group compared to the control group by 35% and 33%, respectively, while the statistical significance of the differences between the group of survivors and the group of the deceased persists (*p* < 0.05 and *p* < 0.001, respectively).

The index with urea and albumin widely discussed in the literature in the group of survivors does not differ from the control, and it significantly increases about fourfold in the group of deceased, being much higher from both the control and the group of survivors (*p* < 0.01 and *p* < 0.001, respectively). Also included in the table is a new index [Urea] × [MDA] × 1000/(BChEb × [ALB]), which may happen to be the most sensitive diagnostic indicator. In the group of deceased, this indicator is 10 times higher than in the group of survivors (*p* < 0.01), and 26 times higher than in the control group of healthy elderly people (*p* < 0.0001).

## 3. Methods

### 3.1. Chemicals

CBDP was synthesized in the Research Institute of Hygiene, Occupational Pathology and Human Ecology with the main component content not less than 95% according to NMR analysis. Deuterated dimethyl sulfoxide (DMSO d6) and deuterated water were provided by the Centre for Magnetic Resonance of St. Petersburg State University. PBS (pH 7.4) was purchased from Biolot (Russia). All other chemicals were purchased from Sigma-Aldrich (St. Louis, MO, USA). Fatty-acid-free HSA and BSA were used.

### 3.2. Patients

The following groups of patients were formed: elderly (60–70 years old) apparently healthy subjects (n = 9), healthy middle-aged people (30–50 years old) practicing open water swimming (n = 12) and elderly people (60–70 years old) with COVID-19 in their history, which were previously divided into two general groups: survivors (n = 19) and deceased (n = 10). In the original cohort, there were 77 survivors and 24 deceased patients aged 30–75 years.

Blood samples were collected, processed and stored according to international consensus guidelines [61].

### 3.3. Assay Methods

An experimental sample of heparinized plasma was thawed at room temperature and spun in an Eppendorf C5810R centrifuge for 30 min, at a rotation speed of 20,000 G and a temperature of 4 °C. After centrifugation, a sample was taken from supernatant and diluted 10 times with PBS 10 mM or TRIS 100 mM (pH = 8.0). Plasma was not diluted for measurement of the activity towards POX, nor for measurement of standard biochemical parameters ([ALB], [Urea], [Creatinine], [TP], [MDA]). All samples were incubated at a constant temperature of 22 °C for 10 min in a BiosanThermo-ShakerTS-100C thermostat.

Protein concentration was measured by the biuret method using BSA as a standard. BChE activity was measured using ATCh and BTCh as substrates in 10 mM PBS. ATCh and BTCh were dissolved in PBS. The method is based on the Ellman reaction [62], namely on the ability of sulfur-containing substrates to form a substance absorbing at 405 nm with 5,5′-dithiobis-(2-nitrobenzoic acid) (DTNB). To prepare 2 mM of DTNB solution, a portion was dissolved in PBS with heating and stirring. The reaction kinetics were recorded spectrophotometrically at 405 nm for 10 min at 37 °C.

Plasma aryl esterase activity was measured using PhAc as a substrate in 100 mM TRIS HCl (pH 8.0) containing 2 mM CaCl_2_. A stock solution of PhAc was dissolved in ethanol. Samples were preincubated with a nonspecific cholinesterase inhibitor, neostigmine methyl sulfate (50 μM), at a constant temperature of 22 °C for 10 min. The reaction kinetics were recorded spectrophotometrically at 270 nm for 10 min at 22 °C.

To measure albumin activity towards NPA, two buffers were used: (1) 10 mM PBS containing 2 mM of EDTA (pH 8.1) and (2) 10 mM PBS containing 2 mM of EDTA and 1 mM of sodium palmitate (pH 8.1). Albumin activity was calculated as the difference between the activities in these media. Preliminary incubation of the sample with a nonspecific cholinesterase inhibitor, neostigmine methyl sulfate (50 μM), was carried out at a constant temperature of 22 °C for 10 min at 37 °C. The reaction kinetics were recorded spectrophotometrically in a plate reader at 405 nm for 10 min.

In the case of BChE activity measurement, the reaction mixture consisted of 5 µL of diluted sample, 185 µL of PBS and 80 µL of developer solution, which contained DTNB (2 mM) and a substrate (70 mM for ATCh or 63 mM for BTCh) mixed in a ratio of 9:1. In the case of PON1 activity measurement, the reaction mixture consisted of 34 µL of diluted sample, 43 µL of neostigmine methyl sulfate, 739 µL of TRIS (100 mM) Ca^2+^ (2 mM) buffer and 860 µL of developer solution, which contained TRIS (100 mM) Ca^2+^ (2 mM) buffer and PhA solution (9.96 mM) mixed in proportions 9:1.

The protein activity in each medium was calculated using Equation (1).

(1) $$A,\;uM\;{min}^{-1}(mM\;{min}^{-1})=\frac{∆absorbance*V}{v_{sample}*\Delta t*\varepsilon *l}*10$$

where *A* is the measured activity; *V* is the volume of the analyzed mixture, μL; *v_sample_* is the volume of the diluted sample, μL; (Δ*E*/Δ*t*) is the change in absorbance in 1 min; *l* in the denominator is the thickness of the absorbing layer; and *ε* is the micromolar extinction coefficient of 2-nitro-5-sulfonylbenzoic acid (0.0145 μM ^−1^ cm ^−1^), phenol (1.31 mM^−1^ cm^−1^) or *p*-nitrophenol (0.01815 μM ^−1^ cm ^−1^). Each sample was measured in triplicate. The blood plasma was diluted 10-fold with the appropriate buffer (PBS 10 mM or TRIS 100 mM). As for the compositions of the analyzed mixtures, in the case of BChE activity, it was 5 µL of a diluted sample, 185 µL of PBS and 80 µL of a developer (coloring) solution, which consisted of 2 mM of DTNB and 70 mM of substrate for ATCh and 63 mM for BTCh, mixed in a ratio of 9:1. When determining PON1 activity, the composition was 34 µL of diluted sample, 43 µL of neostigmine methyl sulfate, 739 µL of TRIS 100 mM with Ca^2+^ 2 mM buffer and 860 µL of developer solution, which consisted of TRIS 100 mM with Ca^2+^ 2 mM buffer and 9.96 mM PhA solution mixed in proportions 9:1. When determining the activity of albumin by 4-NPA, the composition of the reaction mixture was 10 μL of a diluted sample, 6.3 μL of neostigmine methyl sulfate, 103.7 μL of PBS EDTA 2 mM or PBS EDTA 2 mM with palmitate 1 mM and 100 μL of developer solution consisting of appropriate buffer and 100 mM NPA in ethanol mixed 9:1.

### 3.4. Albumin Electrophoresis in Polyacrylamide Gel

Polyacrylamide gel electrophoresis of Sigma-Aldrich BSA sample in the presence of sodium dodecyl sulfate (SDS) was performed using a Mini-Protean electrophoretic chamber (BioRad). Before applying to the gel, a buffer containing 0.125 M of Tris-HCl (pH 6.8), 20% glycerol, 4% SDS and 0.002% bromophenol blue was added to the protein samples. Then, 10% DTT was also added to some of the samples. The concentration of the BSA solution was 4 mg/mL (60 μM). Line 1 was loaded with 3 µL of the BLUelf Prestained Protein Ladder marker (GeneDirex). In lines 3–7, 1, 2, 5, 10 and 15 µL of BSA solution were applied without the addition of DTT. In lines 9–13, 1, 2, 5, 10 and 15 µL of BSA solution with DTT were applied. Gels after electrophoresis were stained with 0.01% Coomassie R-250 solution.

### 3.5. Effect of Inhibitors on the Esterase Activity of Serum Albumin In Vitro According to Spectrophotometry Data

The study of the effect of inhibitors (solutions ethopropazine and CBDP in DMSO) on the esterase activity of BSA towards NPA was carried out in the mode of preliminary incubation of the studied inhibitor with BSA solution during 30 min at t = 25 °C. The concentrations of BSA, ethopropazine, CBDP and NPA in the reaction mixture were 150, 2.5, 0.025 and 3000 µM in experiment 1 and 360, 6, 0.06 and 7800 µM in experiment 2, respectively. Samples of BSA and NPA in PBS, as well as BSA and NPA in PBS with the addition of DMSO (volume equivalent to the volume of inhibitor solutions, 10% of the total volume of the mixture) were used as the controls. The concentration of *p*-nitrophenol (a product of NPA hydrolysis) was determined spectrophotometrically (405 nm) for 15 min. Each sample was measured in triplicate. Primary processing of the obtained data was performed using MS Excel. The change in absorption from 267 to 360 s of the experiment (stationary phase of the reaction) was used for the calculation of BSA relative activity, while the arithmetic mean between the control samples with PBS and DMSO was taken as 100%. The statistical significance of differences was assessed using the Mann–Whitney test.

### 3.6. Effect of Inhibitors on the Esterase Activity of Serum Albumin In Vitro According to NMR Data

On the day of the experiment, a solution of BSA in a mixture of PBS and deuterated water (9:1), a solution of the internal standard DSS in bi-distilled water and solutions of NPA, ethopropazine and CBPD in DMSO d6 were prepared. Solutions of inhibitors and BSA were mixed and incubated for 30 min at room temperature (25 °C). An equivalent volume of DMSO d6 was added to the control sample without inhibitors (thus, the volume of DMSO d6 was identical in each sample, 5% of the total volume of the mixture). Then, immediately before recording the spectrum, the substrate solution was added to the reaction mixture. In the final reaction mixture, the BSA concentration was 360 μM, the NPA concentration was 7.2 mM, the ethopropazine concentration was 240 nM and the CBDP concentration was 24 nM. The prepared samples were scanned by ^1^H NMR in a Bruker Avance III 500 NMR spectrometer at room temperature (25 °C). To suppress the water signal, the excitation sculpting method with “perfect echo” for cleaning phase distortions was used [63]. The spectra were recorded every 3 min. At each time point, 32 scans were performed. The relaxation delay was set to 1 sec. Chemical shifts δ of solution components accurate to 0.001 ppm were calibrated against tetramethylsilane Si(CH_3_)_4_. DMSO was used as the internal standard. The relative integral intensity (I_rel_) of the signals of NPA and the products were calculated as the ratio of their integrated signal intensity to the integrated signal intensity of DMSO d6.

### 3.7. Molecular Modeling Methods

#### 3.7.1. Three-Dimensional Models Preparation

Three-dimensional models of NPA and neostigmine were built and optimized using the steepest-descent method using the HyperChem 8.0 program [64]. The crystal structures of HSA from the Protein Data Bank [65] were used as the three-dimensional models of human albumin, structure codes 2bxd (for docking to Sudlow site I) and 2bxg (for docking to Sudlow site II) [66]. Water and ligand molecules were removed from the structures, and missing atoms were completed using the VMD software package [67].

#### 3.7.2. Molecular Docking

Docking of NPA and neostigmine to the binding sites of HSA was performed using the Autodock Vina 1.1.2 software package [68]. In the studied protein binding site, a search area of 15 × 15 × 15 Å^3^ was set. The number of runs (exhaustiveness) was taken to be equal to 10. The allowable maximum scatter of conformational energies in the output file (energy_range) was taken to be 3 kcal/mol. The number of the most optimal (energetically favorable) conformations in the output file (num_modes) was set to 10. The result of each docking procedure was a set of 10 most energetically favorable conformations. In the case of docking of NPA, we selected the conformation of the protein–ligand complex with the minimum distance between the carbonyl atom of the ligand and the oxygen atom of the hydroxyl group of the catalytic amino acid. In the case of docking of neostigmine, the most energetically favorable conformations were chosen. Two-dimensional diagrams of ligand–protein complexes were drawn using LigPlot+ v.2.2 (European Bioinformatics Institute, United Kingdom) [69].

#### 3.7.3. Molecular Dynamics

Conformational changes in ligand–albumin complexes were calculated by the MD method using the GROMACS2019.4 software [70] and CHARMM27 force field [71]. The complexes were placed virtually into a cubic periodic box filled with water molecules. The TIP3P water model (transferable intermolecular potential with 3 points) was used to describe water molecules [72]. To neutralize a system, sodium ions were added. Temperature (300 K) and pressure (1 bar) were kept constant using a V-rescale thermostat [73] and a Parrinello–Rahman barostat [74], with coupling constants of 0.1 ps and 2.0 ps, respectively. Long-range electrostatic interactions were treated by the particle-mesh Ewald method [75]. Lennard-Jones interactions were calculated with a cutoff of 1.0 nm. The LINCS algorithm was used to constrain bond length [76]. Before running the MD simulations, all the structures were minimized by steepest-descent energy minimization and equilibrated under NVT (1000 ps) and NPT (1000 ps) ensembles. The time step for MD simulation was 0.002 ps.

### 3.8. Statistics

Statistical data processing was carried out using the GraphPad Prism program. For descriptive statistics, mean values with standard deviations or medians with a spread from minimum to maximum were used. Testing for normality of distribution was carried out in several ways: the D’Agostino–Pearson omnibus normality test, the Shapiro–Wilk normality test and the Kolmogorov–Smirnov normality test. To compare the two groups, the Student’s *t*-test (unpaired *t*-test) or the Mann–Whitney test were used, depending on the type of data distribution. To compare several groups, a non-parametric variant of analysis of variance (ANOVA) was used—the Kruskal–Wallis test. In all statistical analyses, the significance level was set to α < 0.05.

## 4. Conclusions

In this paper we attempted to re-evaluate the ideas regarding the esterase status of blood plasma, which is one of the most accessible biological matrices and also the most saturated with biomarkers that are of the greatest value for diagnostics. We studied by in vitro methods the influence of inhibitors on the esterase status of blood plasma, which was taken from apparently healthy subjects. Additionally, we investigated the influence of ethopropazine, CBDP, warfarin and diazepam on esterase activity of commercially available fatty-acid-free albumin. It was shown that CBDP and ethopropazine, in the range of concentrations where they inhibit CE and BChE, do not influence albumin activity. Therefore, new fundamental evidence of the presence of true esterase activity in albumin was obtained. At the same time, an increase in the esterase activity of albumin was noted after the addition of DMSO, which suggests a contribution of Kemp elimination that occurs in the Stern layer to the “apparent” kinetics of both albumin sites. The magnitude of this contribution and the possibilities of its modulation are of paramount interest and deserving of special attention by researchers.

The interaction of neostigmine with Sudlow sites I and II of HSA was studied by molecular modeling methods. It has been established that neostigmine interacts only with Sudlow site I, but the binding of neostigmine does not affect the catalytic Tyr150, so hydrolysis of NPA in this site is still possible, since neostigmine does not affect the interaction of NPA with Sudlow site II.

Our next step was to investigate the esterase status of human blood plasma in healthy people of middle age (swimmers) and elderlies. Reliable differences were found between the groups in creatinine concentration, total protein, MDA and BChE activities with two substrates, while no significant differences were found in albumin and urea levels between the groups. Significant differences were established for ratios of BChE activity to [ALB] and to [MDA], as well as in the ratio of [Urea] × [MDA]/[ALB] and [Urea] × [MDA]/(BChEb × [ALB]).

A comparative analysis of the esterase status of apparently healthy elderly subjects and patients of the same age with COVID-19 at the final stage of our multifaceted study allowed us to obtain additional evidence of the importance of albumin as a diagnostic marker. Relative to the control, the albumin level in the survivors and deceased patients was significantly lower by 18 and 27%, respectively, while the significant differences in total protein were less expressed. The preservation of the protein-synthesizing function of the liver in deceased patients indicates a probable switch of this function to the synthesis of CRP and other positive acute-phase proteins.

The index with urea and albumin, which has already become popular in the scientific community, did not in our study differ from the control in the group of survivors, although in the group of the deceased, it was increased by about four times. Of particular interest is the index [Urea] × [MDA] × 1000/(BChEb × [ALB]), which in the group of deceased was 10 times higher than in the group of survivors and 26 times higher than the value in the group of apparently healthy elderly people. It should be understood that the value of this kind of biochemical index is due to the simplicity and low cost of obtaining or calculating them, which is especially important for laboratories that do not have the ability to conduct expensive analyses with the participation of highly qualified specialists.

Our data based on clinical trials provide grounds for further large-scale clinical trials in accordance with the requirements of evidence-based medicine. As for further research studies of a fundamental nature, they are associated with the need to obtain additional characteristics of the enzymatic activity of albumin in the composition of blood plasma, i.e., first of all, taking into account crowding effects. This is another point for our future research.

## 5. Limitations of the Research

Conceptual work is often carried out outside of mainstream funded research, so it is not possible to completely satisfy all the stringent requirements of evidence-based medicine or even the high-level experimental science. In particular, additional positive and negative controls are necessary with purified and/or recombinant proteins, and with all of their isoforms, in order to prove their presence as possible impurities. Regarding carboxyl esterases in human blood plasma, we need to check at least four enzymes: CES1, CES2, CES3 and ESD. This is another point for our future research.

Particular difficulties are associated with the expansion of the sample of patients, the essence of which is to justify the receipt of additional blood samples and/or additional tests, which is not provided for by the existing standards of clinical diagnostics. On the other hand, none of the multicenter randomized trials involving several thousand people are possible without preliminary small-cohort comparative analysis.

In addition, the reasons should be mentioned for the use of BSA instead of HSA in our experiments in vitro. This article presents in vitro data obtained by NMR and is a continuation of our already-published studies on NMR with BSA as the object of research. From the point of view of the NMR research strategy, we considered it more appropriate to continue the work already begun with BSA than to start these studies with HSA and spend disproportionately more time, as it would be necessary to obtain the basic kinetic characteristics already obtained with BSA.

## Figures and Tables

**Table 1:** Comparison of the esterase status of healthy elderly people and swimmers before exercise. Data are presented as medians with a range from minimum to maximum (Me (min; max)).

	Control Group 1 “Elderlies”	Control Group 2 “Swimmers”
Number of samples	9	12
[ALB], g L^−1^	45.2 (43.7; 48.9)	45.7 (42.5; 51.4)
[Urea], mM	5.8 (4.4; 9.7)	4.5 (2.9; 7.8)
[Creatinine], µM	57.8 (49.4; 62.3)	82.5 (68.0; 104.0) ****
[TP], g L^−1^	73.5 (67.9; 80.2)	79.7 (73.2; 86.7) **
[MDA], µM	1.10 (0.60; 1.30)	0.80 (0.33; 2.30) *
BChEa, µM min^−1^	1272 (1015; 1602)	1572 (1189; 2051) *
BChEb, µM min^−1^	2182 (1270; 3358)	3060 (2274; 3953) **
ALBn, µM min^−1^	37.2 (21.7; 70.9)	47.8 (6.0; 186.2)
PON1, mM min^−1^	40.0 (24.6; 51.0)	44.2 (28.9; 55.3)
**Indices**		
[ALB]/[TP]	0.62 (0.59; 0.66)	0.58 (0.50; 0.67) *
[Urea] × 10/[ALB]	1.34 (0.93; 2.14)	0.95 (0.58; 1.71)
[MDA]/[ALB]	2.53 (1.41; 2.94)	1.72 (0.73; 5.27)
BChEa/[ALB]	28.2 (22.5; 32.7)	34.6 (27.1; 46.6) **
BChEb/[ALB]	48.8 (26.0; 74.5)	66.7 (52.0; 87.9) **
[Creatinine] × [MDA]/[ALB]	1.29 (0.81; 1.81)	1.40 (0.76; 3.58)
[Urea] × [MDA]/[ALB]	0.15 (0.09; 0.21)	0.08 (0.03; 0.23) *
BChEa/[MDA]	1144 (870; 1999)	1987 (516; 4024) **
BChEb/[MDA]	1908 (1255; 4282)	3789 (987; 7265) *
BChEa/ALBn	28.5 (17.9; 61.6)	23.8 (10.3; 110.7)
BChEb/ALBn	45.2 (35.2; 154.5)	45.3 (18.8; 199.8)
[Urea] × [MDA] × 1000/(BChEb × [ALB])	0.06 (0.04; 0.12)	0.02 (0.01; 0.10) **

[ALB], concentration of albumin; ALBn, activity of albumin towards NPA; BChEa, activity of BChE towards ATCh; BChEb, activity of BChE towards BTCh; [MDA], concentration of malondialdehyde; PON1, paraoxonase-1; [TP], concentration of total protein. Significant difference between two groups is indicated with asterisks; *, *p* < 0.05; **, *p* < 0.01; ****, *p* < 0.0001.

**Table 2:** Biochemical parameters for surviving and deceased patients with COVID-19, presented in median view with minimum and maximum values (Me (min; max)).

Parameter	Survivors	Non-Survivors
Number of samples, n	19	10
[ALB], g L^−1^	37.0 (29.0; 43.0)	32.7 (25.0; 38.9) *
[TP], g L^−1^	67.7 (58.8; 82.1)	65.1 (48.9; 82.2)
[ALB]/[TP]	0.54 (0.42; 0.66)	0.52 (0.40; 0.58)
[MDA], μM	3.59 (0.74; 7.87)	5.72 (2.12; 8.37)
BChEa, μM min^−1^	1177 (798; 1528)	832 (350; 1535) *
BChEb, μM min^−1^	2435 (1515; 2890)	1471 (850; 2372) ****
ALBn, μM min^−1^	48.3 (6.0; 113.3)	43.0 (6.0; 223.5)
PON1, mM min^−1^	25.8 (1.3; 48.6)	25.9 (11.8; 32.9)
Indices		
[Urea] × 10/[ALB]	1.29 (0.91; 3.44)	5.40 (1.62; 20.20) ****
[MDA]/[ALB]	10.98 (2.00; 25.56)	18.78 (7.29; 27.14) *
BChEa/[ALB]	30.73 (22.61; 46.85)	24.90 (12.38; 39.47)
BChEb/[ALB]	64.01 (40.94; 89.35)	46.85 (25.51; 60.97) ***
[Creatinine] × [MDA]/[ALB]	11.06 (1.36; 26.25)	19.37 (2.14; 53.08)
[Urea] × [MDA]/[ALB]	0.42 (0.07; 1.92)	2.48 (0.54; 13.71) ***
BChEa/[MDA]	293 (101; 1741)	161 (66; 374) *
BChEb/[MDA]	571 (251; 3295)	261 (195; 590) ***
[Urea] × [MDA] × 1000/(BChEb × [ALB])	0.18 (0.03; 0.96)	1.60 (0.56; 10.36) ****

Significant difference between two groups is indicated with asterisks; *, *p* < 0.05; ***, *p* < 0.01; ****, *p* < 0.0001. The Mann–Whitney test was used to assess statistical significance.

**Table 3:** Esterase profile of patients with COVID-19, compared with relatively healthy elderly people (control group, the “elderlies”). The data are presented in the form of medians (Me (min; max)).

	Control Group	Survivors	Non-Survivors
Number of samples	9	19	10
[ALB], g L^−1^	45.2 (43.7; 48.9)	37.0 (29.0; 43.0) ***	32.7 (25.0; 38.9) ****
[TP], g L^−1^	73.5 (67.9; 80.2)	67.7 (58.8; 82.1)	65.1 (48.9; 82.2) *
[MDA], μM	1.1 (0.6; 1.3)	3.59 (0.74; 7.87) *	5.72 (2.12; 8.37) ***
BChEa, μM min^−1^	1272 (1015; 1602)	1177 (798; 1528)	832 (350; 1535) * #
BChEb, μM min^−1^	2182 (1270; 3358)	2435 (1515; 2890)	1471 (850; 2372) * ###
ALBn, μM min^−1^	37.2 (21.7; 70.9)	48.3 (6.0; 113.3)	43.0 (6.0; 223.5)
PON1, mM min^−1^	40.0 (24.6; 51.0)	25.8 (1.28; 48.6) **	25.9 (11.8; 32.6) *
[Urea] × 10/[ALB]	1.34 (0.93; 2.14)	1.29 (0.91; 3.44)	5.4 (1.62; 20.2) **, ###
[Urea] × [MDA] × 1000/(BChEb × [ALB])	0.06 (0.04; 0.12)	0.19 (0.03; 0.96)	1.60 (0.56; 10.36) ****, ##

*, **, ***, ****—differences from the control (elderlies) are statistically significant (*p* < 0.05, *p* < 0.01, *p* < 0.001, *p* < 0.0001, respectively); #, ##, ###—differences between survivors and deceased are statistically significant (*p* < 0.05, *p* < 0.01, *p* < 0.001, respectively).

## Data Availability

The data presented in this study are available from the corresponding authors upon reasonable request.

## Supplementary Materials

The following supporting information can be downloaded at: https://www.mdpi.com/article/10.3390/ijms241210383/s1.

## Abbreviations

AChE	acetylcholinesterase
ALB	concentration of albumin
ALBn	activity of albumin towards p-nitrophenyl acetate
ATCh	acetylthiocholine
BChE	butyrylcholinesterase
BChEa	activity of BChE towards acetylthiocholine
BChEb	activity of BChE towards butyrylthiocholine
BMI	body mass index
BSA	bovine serum albumin
BTCh	butyrylthiocholine
BW284C51	4-(5-{4-[Dimethyl(prop-2-enyl)ammonio]phenyl}-3-oxopentyl)-N,N-dimethyl-N-prop-2-enylbenzenaminium
CBDP	2-(2-cresyl)-4H-1-3-2- benzodioxaphosphorin-2-oxide
CE	carboxylesterase
CK_MB	creatine kinase of myocardial band
CK_NAC	NAC-activated creatine kinase
Crea	creatinine
CRP	C-reactive protein
DMSO	dimethyl sulfoxide
DTNB	5,5′-dithiobis-(2-nitrobenzoic acid)
DTT	dithiothreitol
EDTA	ethylenediaminetetraacetate
ESD	esterase D
FA1-7	fatty-acid binding sites 1-7 in albumin
FRUC	fructosamine
GHSR1	growth hormone secretagogue receptor 1
HSA	human serum albumin
LDL	low-density lipoproteins
LINCS	linear constraint solver
MD	molecular dynamics
MDA	malondialdehyde
NEFA	non-esterified fatty acids
NEO	neostigmine
NMR	nuclear magnetic resonance
NPA	*p*-nitrophenyl acetate
NTE	neuropathy target esterase
NVT and NPT ensembles	constant volume and pressure ensembles
OPs	organophosphates
PDB	Protein Data Bank
PhA	phenyl acetate
PON1	paraoxonase 1
POX	paraoxon
SDS	sodium dodecyl sulfate
SFA	subcutaneous fat area
TP	total protein
TRIGS	triglycerides
Urea	urea
VFA	visceral fat area
