# Supplementary material for: Albumin Is a Component of the Esterase Status of Human Blood Plasma
Source: Int J Mol Sci. 2023 Jun 20;24(12):10383. doi: 10.3390/ijms241210383 (PMC10299176; doi:10.3390/ijms241210383)
Supplement: Supplementary file 1 [file ijms-24-10383-s001.zip › ijms-2280941-SI.pdf]

Supplement

The effect of sequential addition of inhibitors on the esterase status of plasma of healthy humans

Table S1. Activity of pooled plasma towards acetylthiocholine (ATCh)

**Inhibitors**	**Activity after inhibitor(s) addition, μM min^{-1}**	
	**towards ATCh 2 mM (M±SD)**	**towards BTCh 1.87 mM (M±SD)**
control	1369 ± 122	3007 ± 109
BW284c51 (10 μM)	1377 ± 43	3004 ± 119
neostigmine (50 μM)	38 ± 21	66 ± 6
neostigmine (50 μM) + EDTA (2 mM)	58.0 ± 14	83 ± 35
neostigmine (50 μM) + EDTA (2 mM) + CBDP (1 nM)	101 ± 69	167 ± 45

EDTA, ethylenediaminetetraacetate; CBDP, 2-(2-cresyl)-4*H*-1-3-2- benzodioxaphosphorin-2-oxide. Control consists an equivalent volume of PBS 10 mM. Plasma was pooled from 10 samples of apparently healthy people. An equivalent amount of an individual sample was taken and mixed in a tube to level out individual characteristics. The statistical significance of differences in activity is shown in Figures 1 and 2 (Kruskal-Wallis test, Dunn's post hoc test, n=8). The plasma was used at 1:10 dilution.

Table S2. Activity of pooled plasma towards 4-nitrophenylacetate (NPA)

**Inhibitors**	**Activity, towards NPA 4.55 mM, μM min $^{-1}$, (M±SD)**
control	1178 ± 141
bw284c51 (10 μM)	1161 ± 159
neostigmine (50 μM)	986 ± 91
neostigmine (50 μM) + EDTA (2 mM)	1307± 101
neostigmine (50 μM) + EDTA (2 mM) + CBDP (1 nM)	1680 ± 70
control + palmitate (1 mM)	1112 ± 49
palmitate (1 mM) + neo (50 μM) + EDTA (2 mM)	1043 ± 63
palmitate 1 mM + neo (50 μM) + EDTA (2 mM) + CBDP (1 nM)	1200 ± 55

EDTA, ethylenediaminetetraacetate; CBDP, 2-(2-cresyl)-4*H*-1-3-2- benzodioxaphosphorin-2-oxide. Controls consist an equivalent volume of PBS 10 mM. Plasma was pooled from 10 samples of apparently healthy people. An equivalent amount of an individual sample was taken and mixed in a tube to level out individual characteristics. The statistical significance of differences in activity is shown in Figure 3 (Kruskal-Wallis test, Dunn's post hoc test, n=4). The plasma was used at 1:10 dilution.

Table S3. Activity of pooled plasma towards phenyl acetate (PhA) with inhibitors of cholin- and carboxyl esterases. The plasma was used at 1:10 dilution.

**inhibitors**	**Activity towards PhA, mM min $^{-1}$**	
	**Control**	**after inhibitor(s) addition**
neostigmine (50 μM)	42.4 ± 2.2	39.9 ± 3.2
neostigmine (50 μM) + CBDP (10 nM)	42.4 ± 2.2	41.3 ± 1.0

*, the level of significance of the change in activity after the addition of inhibitors compared to the control $p < 0.05$

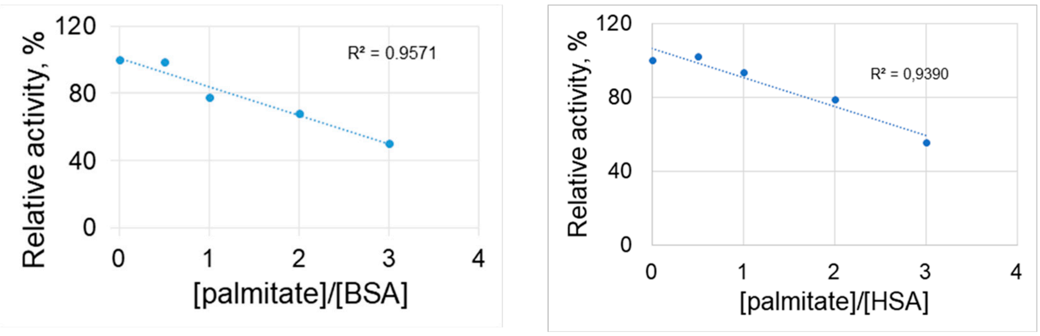

Figure S1. Inhibition by palmitate of pseudoesterase activity of (a) bovine serum albumin (BSA, 10 μM) and (b) human serum albumin (HSA, 10 μM) towards *p*-nitrophenyl acetate (NPA, 20 μM). Activities of fatty acid free BSA and HSA were set as 100%.

Interaction of neostigmine with Sudlow sites I and II of human serum albumin (HSA)

The result of molecular docking of neostigmine to Sudlow site I of HSA is shown in Figure S2A. Sudlow site I is a flexible multi-chamber cavity and consists of a central zone associated with three separate cavities, one of which corresponds to fatty acid binding site FA7. The inner part of the site is predominantly nonpolar, but contains two polar regions: one at the base of the site (Tyr150, His242, Arg257), and the other at its entrance (Lys195, Lys199, Arg218, Arg222). According to molecular docking data, neostigmine binds in the central zone of Sudlow site I. The carboxylic oxygen of neostigmine forms a hydrogen bond with one of the hydrogen atoms of Arg222 guanidine group (Figure S2A). However, according to results of MD simulation, the conformation obtained by molecular docking is unstable. During the simulation process, the conformation of Sudlow site I changes and the neostigmine molecule moves to that part of Sudlow site I that corresponds to FA7 (Figure S2B).

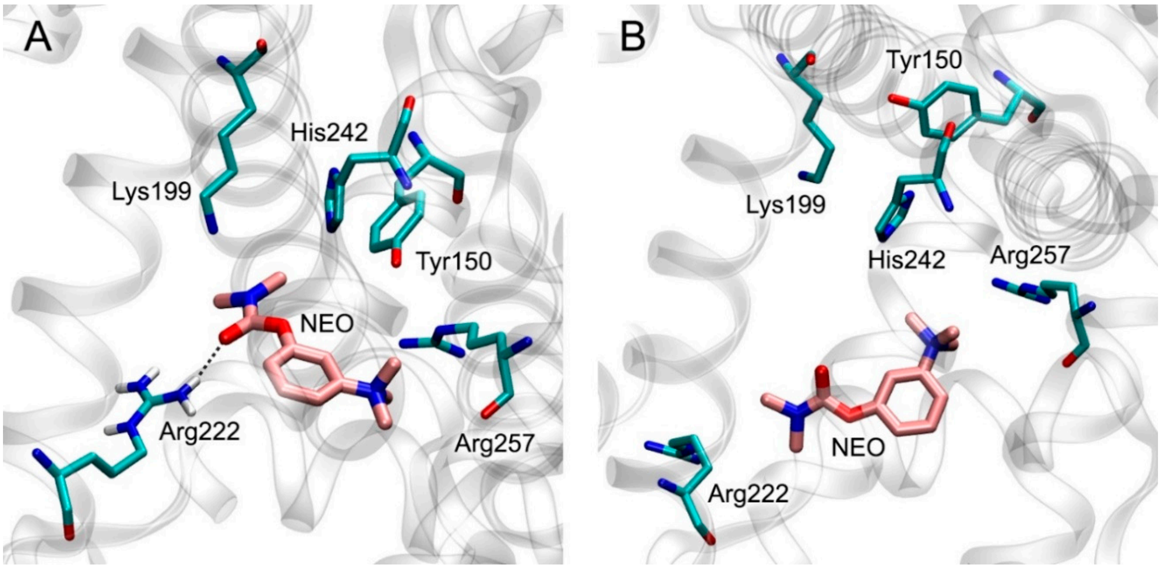

Figure S2. Interaction of neostigmine with Sudlow site I of human serum albumin (HSA) according to molecular docking (A) and molecular dynamics (MD) simulation (B). Non-essential hydrogens are omitted for clarity.

The result of molecular docking of neostigmine to Sudlow site II of HSA is shown in Figure S3A. Sudlow site II of HSA is a large nonpolar pocket (corresponding to fatty acid binding sites FA3 and FA4) with a single polar region, which is formed by Arg410, Tyr411, and Lys414 residues, and is located at the very entrance to the site. Ligands of Sudlow site II, e.g. ibuprofen and diazepam, typically contain a peripheral negative charge. According to molecular docking data, neostigmine binds at the entry of the site near polar amino acids Arg410, Tyr411, and Lys414. The carboxylic oxygen of neostigmine forms hydrogen bonds with the polar hydrogen atoms of the side chains of Arg410 and Lys414. The positively charged trimethylammonium group of neostigmine is localized in the environment of nonpolar amino acids of the site (Ile388, Leu453, Val433, Phe403). But, as in the case of Sudlow site I, according to MD simulation, the conformation obtained by molecular docking is unstable. During the first 2 ns of simulation, the neostigmine molecule leaves Sudlow site II (Figure S3B) and stays in an unbound state for the remaining 98 ns of simulation.

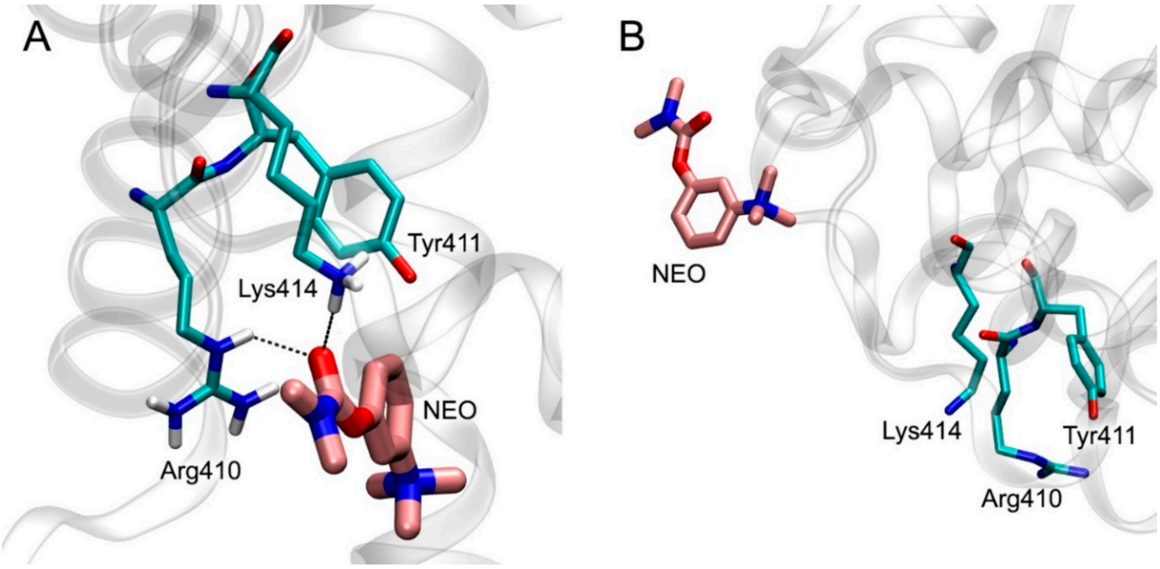

Figure S3. Interaction of neostigmine with Sudlow site II of HSA according to molecular docking (A) and MD simulation (B). Non-essential hydrogens are omitted for clarity.

Esterase status of human plasma in normal and pathological conditions

Table S4. Spearman's rank correlation coefficients of the dependence of albumin concentration and esterase status indicators of patients.

**Esterase status parameter**	**Coefficient of correlation**	**Coefficient of correlation** (Survivors and non-survivors)
MDA, μM	-0.20	-0.12 survivors,<br>0.018 non-survivors
BChEa, μM min^{-1}	0.55**	0.33 survivors,<br>0.59 non-survivors, though without statistical significance because of few observations
BChEb, μM min^{-1}	0.47**	0.13 survivors,<br>0.51 non-survivors, though without statistical significance because of few observations
ALBn, μM min^{-1}	-0.04	-0.16 survivors,<br>0.27 non-survivors
PON1, mM min^{-1}	0.22	0.40 survivors,<br>-0.018 non-survivors
